# Berberine Protects against TNF-*α*-Induced Injury of Human Umbilical Vein Endothelial Cells via the AMPK/NF-*κ*B/YY1 Signaling Pathway

**DOI:** 10.1155/2021/6518355

**Published:** 2021-12-31

**Authors:** Li Chen, Xiao-Di Fan, Hua Qu, Rui-Na Bai, Da-Zhuo Shi

**Affiliations:** ^1^Peking University Traditional Chinese Medicine Clinical Medical School (Xiyuan), Beijing 100191, China; ^2^Department of Integration of Chinese and Western Medicine, School of Basic Medical Sciences, Peking University, Beijing 100191, China; ^3^National Clinical Research Center for Chinese Medicine Cardiology, Xiyuan Hospital of China Academy of Chinese Medical Sciences, Beijing 100091, China; ^4^Institute of Basic Medical Sciences, Xiyuan Hospital of China Academy of Chinese Medical Sciences, Beijing 100091, China

## Abstract

Endothelial injury, characterized by an inflammatory response and increased permeability, is an initial stage of atherosclerosis (AS). Adenosine 5′-monophosphate (AMP), activated protein kinase (AMPK), and Nuclear Factor kappa B (NF-*κ*B)/Yin Yang 1(YY1) signaling pathways play important roles in the process of endothelial injury. Berberine (BBR), a bioactive alkaloid isolated from several herbal substances, possesses multiple pharmacological effects, including anti-inflammatory, antimicrobial, antidiabetic, anticancer, and antioxidant activities. Previous studies showed a protective effect of berberine against endothelial injury. However, the underlying mechanism remains unclear. We explored the potential effect of BBR on TNF- (tumor necrosis factor-) *α*-induced injury of human umbilical endothelial cells (HUVECs) and studied its possible molecular mechanism. In the present study, HUVECs were divided into three groups. HUVEC viability was measured with Cell Counting Kit-8 assay. Extracellular lactic dehydrogenase (LDH) concentration was measured with LDH leakage assay. Endothelial microparticle (EMP) numbers were evaluated by flow cytometry analysis assay. The expression of proinflammatory cytokines was evaluated by Enzyme-Linked Immunosorbent Assay (ELISA). The mRNA expression of NF-*κ*B and YY1 was detected by Real-Time PCR (RT-PCR). The protein expression of NF-*κ*B, YY1, and AMPK was detected by immunofluorescence microscopy assay or western blot analysis. The results showed that LDH concentration, EMPs numbers, and the expression of proinflammatory cytokines (IL-6, IL-8, and IL-1*β*) increased in TNF-*α*-induced injured HUVECs, but ameliorated by BBR pretreatment. BBR pretreatment upregulated the expression of phosphorylated AMPK and downregulated the expressions of NF-*κ*B and YY1 in injured HUVECs induced by TNF-*α*, which were offset by the AMPK inhibitor Compound C (CC). The results indicated that BBR protected against TNF-*α*-induced endothelial injury via the AMPK/NF-*κ*B/YY1 signaling pathway.

## 1. Introduction

Atherosclerosis, a progressive inflammatory disease of large- and medium-sized arteries, is the main pathological changes of cardiovascular diseases [1]. Endothelial cell dysfunction, manifested in lesion-prone areas of the arterial vasculature, results in the earliest detectable changes in the life history of an atherosclerotic lesion the focal permeation, trapping, and physicochemical modification of circulating lipoprotein particles in the subendothelial space [2]. Endothelial injury, characterized by an inflammatory response and increased permeability, is an initial stage of atherosclerosis (AS). Increased endothelial permeability leads to augment of reactive oxygen species (ROS), deposition of low-density lipoproteins (LDLs), and infiltration of circulating leucocytes [3–5]. Extracellular lactic dehydrogenase (LDH) concentration and endothelial microparticle (EMP) numbers are the main indices of endothelial permeability [6, 7]. The expression of inflammatory cytokines, including interleukin 6 (IL-6), IL-8, and IL-1*β*, promotes the adhesion and infiltration of monocytes into the vascular endothelium and further leads to endothelial injury [8–10]. Moreover, the inflammatory response causes dissociation of cell-cell junctions between endothelial cells as well as cytoskeleton contraction, resulting in endothelial permeability [11]. The hyperpermeability of the endothelium, in turn, contributes to the vascular inflammatory response by activating inflammatory cytokines [12–14].

Adenosine 5′-monophosphate (AMP) activated protein kinase (AMPK) is an enzyme mainly regulated by cellular AMP and plays a vital role during the period of energy stress which can restore energy balance by phosphorylating multiple key factors [15, 16]. AMPK activation is involved in many inflammatory signaling pathways and closely associated with atherosclerosis [17]. NF-*κ*B has long been considered a prototypical proinflammatory signaling pathway, largely based on the activation of NF-*κ*B by proinflammatory cytokines such as IL-6 and tumor necrosis factor *α* (TNF*α*) and the role of NF-*κ*B in the expression of other proinflammatory genes including cytokines, chemokines, and adhesion molecules [18, 19]. NF-*κ*B activation aggravates the inflammatory response by upregulating inflammatory cytokines [20]. Yin Yang 1 (YY1), a 65-kDa ubiquitous multifunction transcription factor, is a member of the GLI-Kruppel family and regulates inflammatory gene expression via binding to the NF-*κ*B p65 heterodimer complex promoter context [21, 22]. Previous studies demonstrated that the NF-*κ*B/YY1 signaling pathway is closely associated with endothelial injury [22–24].

Berberine (BBR), the principal component of *Coptis chinensis* Franch. (family Ranunculaceae) [25], is a main active ingredient in plenty of prescriptions including Huanglian-Jie-Du-Tang (decoction of Coptidis rhizoma, Scutellariae radix, Phellodendri cortex, and Gardeniae fructus), DaHuang-Haunglian-Xie-Xin-Tang (Rhubarb and Coptis Heart-Draining Decoction), and Huanglian Tang (Coptis Decoction), which have been used in clinical treatment for centuries [26]. Nowadays, BBR is used extensively to treat certain diseases, such as type-2 diabetes mellitus (T2DM) [27–29], neurodegenerative diseases [30, 31], tumors [32–34], and atherosclerotic diseases [35–37]. Whether BBR protects against endothelial injury via the AMPK/NF-*κ*B/YY1 signaling pathway remains unclear. This study was designed to test the effect of BBR on protecting against endothelial injury and explore its possible mechanism. Preliminary experiments demonstrated that 20 *μ*M BBR significantly increased HUVEC viability injured by TNF-*α*(*P* < 0.01), so the concentration of BBR was chosen as 20 *μ*M in the present study.

## 2. Materials and Methods

### 2.1. Cell Culture

HUVECs (ScienCell, USA, #8000) were cultured in an endothelial cell medium (ECM) with 5% FBS, 1% endothelial cell growth supplement, and 1% penicillin/streptomycin at 37°C in a humidified atmosphere with 5%CO_2_. HUVECs within 4–7 passages were used and divided into three groups: (1) control group: HUVECs were cultured in the ECM medium without serum for 24 h and then in the ECM medium with serum for 24 hours; (2) TNF-*α* group: HUVECs were cultured with no serum for 24 hours and then in the ECM medium with serum and TNF-*α* 20 ng/mL for 24 hours; and (3) TNF-*α*+BBR (20 *μ*M) group: beside the 2 groups, HUVECs were pretreated with 20 *μ*M BBR for 2 hours.

### 2.2. Chemical Reagents and Antibodies

Recombinant human TNF-*α* was purchased from PeproTech Inc. (Rocky Hill, NJ, USA, Lot: 300–01A). TNF-*α* was dissolved in ultrapure water and stored in −20°C. Endothelial cell medium, fetal bovine serum, endothelial cell growth supplement, and penicillin/streptomycin solution (#1001) were purchased from ScienCell Research Laboratories (Carlsbad, CA, USA¸ Lot: #8000). Berberine HPLC ≥ 98% was purchased from Coolaber Science and Technology (China, Lot: Z21449). Berberine was dissolved in dimethyl sulfoxide and stored in −20°C. Anti-AMPK antibody (2532s) and phosphor-AMPK antibody (2535T) were purchased from Cell Signaling Technology. Anti-NF-*κ*B p65 antibody (ab32536), anti-YY1 antibody (ab109237), and goat anti-rabbit IgG H&L (ab150077) were purchased from Abcam Biotechnology. IL-1*β* Rabbit Polyclonal antibody was purchased from Proteintech (China, Lot: 16806-1-AP). Compound C, 6-[4-(2-piperidin-1-yl-ethoxy)-phenyl)]-3-pyridin-4-yl-pyrrazolo-[1,5-a]-pyrimidine), was purchased from Sigma-Aldrich (St. Louis, MO, USA, Lot: CAS866405-64-3). Peroxidase-conjugated goat anti-rabbit lgG (H + L) was purchased from ZSGB-BIO. PE mouse anti-human CD31 and APC mouse anti-human CD42b were purchased from BD Bioscience (Becton, Dickinson and Company, USA, Lot: 566125, 551061). CCK-8 assay kits were purchased from Dojindo Molecular Technologies (Japan, Lot: CK04). The LDH cytotoxicity assay kit and BCA protein assay kit were purchased from Beyotime (China, Lot: C0017, BC201). Human IL-6 and IL-8 ELISA kits were purchased from Multisciences (China, Lot: EK1062 and EK1082). RIPA was purchased from Solarbio Life Science (China, Lot: P0013B). TRIzol reagent was purchased from Invitrogen (Sigma, USA, Lot: 15596018).

### 2.3. Cell Viability Assay

HUVECs were seeded in 96-well plates with the density of 6** × **10^3^ per well and cultured in a medium (including 5% FBS, 1% endothelial cell growth supplement, and 1% penicillin/streptomycin) for 24 hours. The medium was replaced without serum for 24 h incubation. HUVECs were subsequently treated with different concentrations of TNF-*α* or BBR for 24 hours. Thereafter, HUVEC viability was measured with Cell Counting Kit-8 assay according to the instructions of the manufacturer. The absorbance at 450 nm was detected using a microplate reader.

### 2.4. LDH Leakage

HUVECs were pretreated for 2 hours with BBR (20 *μ*M and 40 *μ*M) and then stimulated with TNF-*α* (20 ng/mL) for 24 hours. The 120 *μ*L supernatant of each well was taken out and transferred into new 96-well plates with 60 *μ*L LDH testing fluid and incubated at room temperature (about 25°C) for 30 minutes. The absorbance at 490 nm was detected using a microplate reader.

### 2.5. Flow Cytometry Analysis

The supernatant was centrifuged at 5000 **×** g for 10 minutes and at 20500 **×** g for 150 minutes for collecting EMPs. The EMPs were washed in PBS and incubated with 10 *μ*L of PE mouse anti-human CD31 and 5 *μ*L APC mouse anti-human CD42b for 1 hour at 4°C. EMPs were then washed three times in PBS and detected with a Coulter EPICS XL flow cytometer (Beckman Coulter, Villepinte, France). Finally, EMPs of 0.1 to 1 um diameter were calculated with an FL/FSC fluorescence dot plot.

### 2.6. Enzyme-Linked Immunosorbent Assay (ELISA)

The supernatant was collected to test the expression of IL-6 and IL-8 by ELISA according the manufacturer's instructions. Briefly, combining rat anti-mouse cytokine antibodies with IL-6 and IL-8, the absorbance at 450 nm was detected using a microplate reader.

### 2.7. Real-Time PCR (RT-PCR)

Total RNA was extracted using TRIzol according to the manufacturer's instructions. cDNA was diluted to a concentration of 1.5 *µ*M. The RT-PCR was performed by using a Bio-Rad MyIQ single-color RT-PCR detection system with Optic system software version 1.0. Ten ng cDNA, 2 x universal PCR master mix, 300 nmol/L forward primer, 300 nmol/L backward primer, and 200 nmol/L Taqman probe were mixed for each PCR, and the final volume was 25 *µ*L. PCR amplification of the housekeeping gene cyclophilin A and of NF-*κ*B and YY1 was performed (1 cycle at 50°C for 2 minutes and 1 cycle at 95°C for 10 minutes, followed by 50 cycles of 95°C for 15 seconds and 60°C for 1 minute). A standard curve was generated, and all assays were performed in duplicate. Relative RNA copy numbers were calculated from standard curves that were obtained by serial dilution of quantified template cDNA. The relative expressions of NF-*κ*B and YY1 were calculated using the 2^−△△Ct^ method. Fold change = 2^−△△Ct^, △△Ct = (Ct _Sample_ − Ct _*β*-actin_)−(Ct _Control_ − Ct _*β*-actin_). The expression of each target gene was normalized referring to the expression of the housekeeping gene cyclophilin. The primers used in RT-PCR are shown in Table 1.

### 2.8. Immunofluorescence Microscopy

HUVEs were washed three times after fixing with 4% paraformaldehyde and incubated with primary antibodies, including anti-NF-*κ*B p65 antibody and and anti-YY1 antibody, at 1 : 100 dilution for overnight at 4°C, and incubated with secondary antibody, goat anti-rabbit lgG (Alexa Fluor 488), at 1 : 1000 dilution for 2 h with 4′-6-diamidino-2-phenylindole (DAPI) nuclear counterstain. HUVECs were then pictured with a confocal microscope (ZEISS, Germany) and analyzed using ZEN microsystem software.

### 2.9. Western Blot Analysis

The proteins were extracted from HUVECs using RIPA buffer with 1% protease inhibitors and 1% phenylmethanesulfonyl fluoride (PMSF). After measurement of protein concentration, the equivalent amounts of protein (10 *μ*L/lane) were separated by 8–12% sodium dodecylsulfate-polyacrylamide gels for electrophoresis and electrophoretically transferred to polyvinylidene difluoride membranes. The membranes were blocked with 5% skimmed milk at room temperature for 2 hours and then incubated with primary antibodies overnight at 4°C, followed by incubation with secondary antibodies at room temperature for 1 hour. The immunoreactive bands were then visualized using the SuperSignal West Femto Maximum Sensitivity Substrate reagent (Thermo Scientific). Rabbit anti-*β*-actin was used as an inner control. The images were quantitatively analyzed using the Image J program.

### 2.10. Statistical Analysis

All statistical analysis was performed using GraphPad Prism version 5.0 (GraphPad software, San Diego, CA, USA). The data were presented as mean ± SD (standard deviation), and the difference between groups was compared using one-way analysis of variance (ANOVA) and Student's *t*-test. Values of *P* < 0.05 were considered statistically significant.

## 3. Results

### 3.1. Berberine Improves the Viability of HUVECs

**Figure 1:**
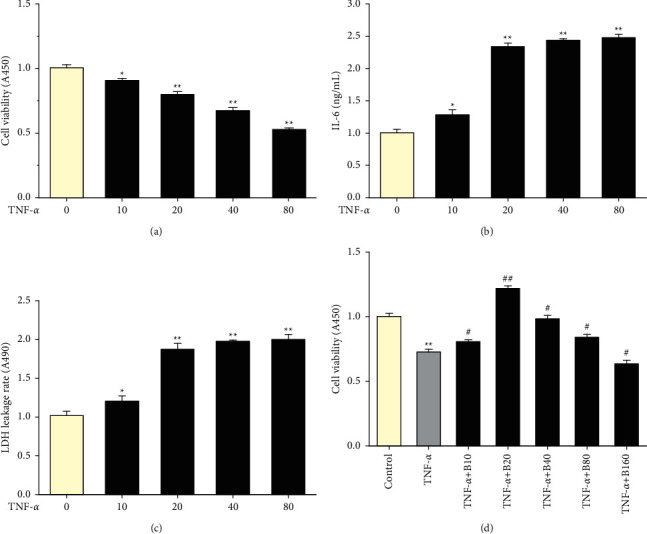
Viability of HUVECs, IL-6 expression, and LDH leakage in TNF-*α*-induced injured HUVECs. (a) HUVECs viability. (b) IL-6 expression. (c) Extracellular LDH concentrations. (d) HUVEC viability after pretreatment with BBR for 2 hours followed by TNF-*α* (20 ng/mL) for 24 hours. n = 3. Data are expressed as mean ± SD. ^*∗*^*P* < 0.05 and ^*∗∗*^*P* < 0.01 compared with the control group. #*P* < 0.05 and ##*P* < 0.01 compared with the TNF-*α* group.

To assess the effect of BBR pretreatment on the viability of HUVECs, CCK-8 assays were performed. After incubation with TNF-*α* at the concentration of 20 ng/mL for 24 h, the cell viability was decreased (*P* < 0.01, Figure 1(a)), and the levels of proinflammatory cytokine (IL-6) and extracellular LDH concentration were increased as compared with the control group (*P* < 0.01, Figures 1(b) and 1(c)). However, pretreatment with 20 *μ*M BBR significantly increased the viability of HUVECs compared with the TNF-*α* group (*P* < 0.01, Figure 1(d)).

### 3.2. Berberine Reduces the Endothelial Permeability of HUVECs

**Figure 2:**
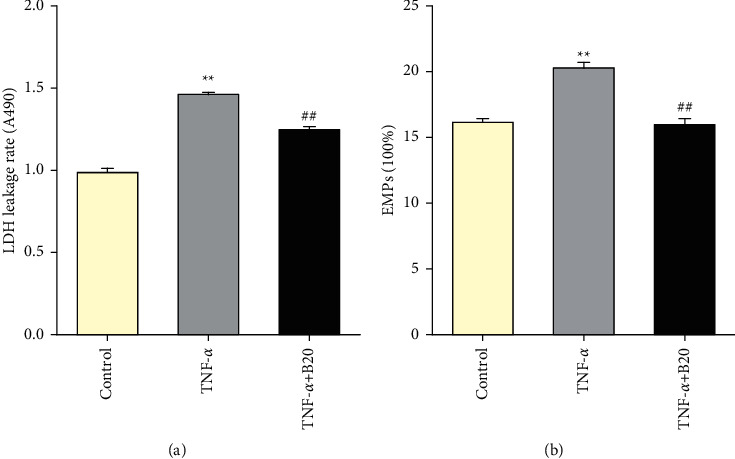
Effects of BBR on extracellular LDH concentration and EMPs numbers in TNF-*α*-induced injured HUVECs. (a) LDH concentration. (b) EMP numbers. Data are expressed as mean ± SD. *n* = 3. ^*∗*^*P* < 0.05 and ^*∗∗*^*P* < 0.01 compared with the control group. #*P* < 0.05 and ##*P* < 0.01 compared with the TNF-*α* group.

Extracellular LDH concentration and EMPs numbers were measured to test the effect of BBR pretreatment on endothelial permeability. After incubation with TNF-*α* at a concentration of 20 ng/mL for 24 hours, extracellular LDH concentration (*P* < 0.01 vs. control) and EMP numbers (*P* < 0.01 vs. control) increased as compared with the control group. As compared with the TNF-*α* group, BBR pretreatment decreased extracellular LDH concentration (*P* < 0.01, Figure 2(a)) and EMP numbers (*P* < 0.01, Figure 2(b)).

### 3.3. Berberine Decreases the Expression of Proinflammatory Cytokines

**Figure 3:**
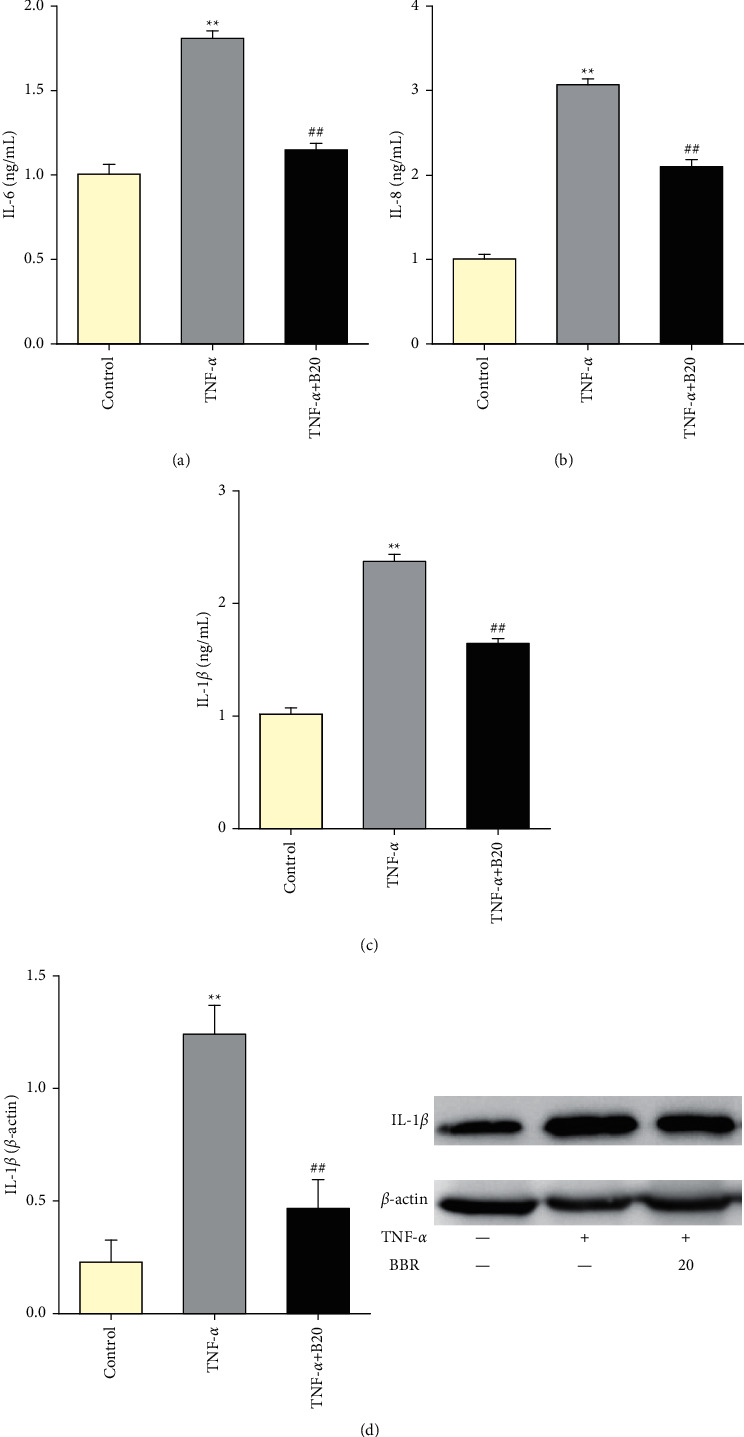
Effect of BBR on the expression of IL-6, IL-8, and IL-1*β* in TNF-*α*-induced injured HUVECs. (a) IL-6 expression. (b) IL-8 expression. (c-d) IL-1*β* expression. Data are expressed as mean ± SD. *n* = 3. ^*∗∗*^*P* < 0.01 compared with the control group; #*P* < 0.05 and ##*P* < 0.01 compared with the TNF-*α* group.

IL-6, IL-8, and IL-1*β* were detected to investigate the effect of BBR pretreatment on TNF-*α*-induced inflammation. After incubation with TNF-*α* at the concentration of 20 ng/mL for 24 hours, the expression of IL-6, IL-8, and IL-1*β* increased (*P* < 0.01 vs. control). Compared with the TNF-*α* group, BBR pretreatment at concentration of 20 *μ*M decreased the expressions of IL-6, IL-8, and IL-1*β* (*P* < 0.05, Figure 3).

### 3.4. Berberine Decreases the Expression of NF-*κ*B and YY1

**Figure 4:**
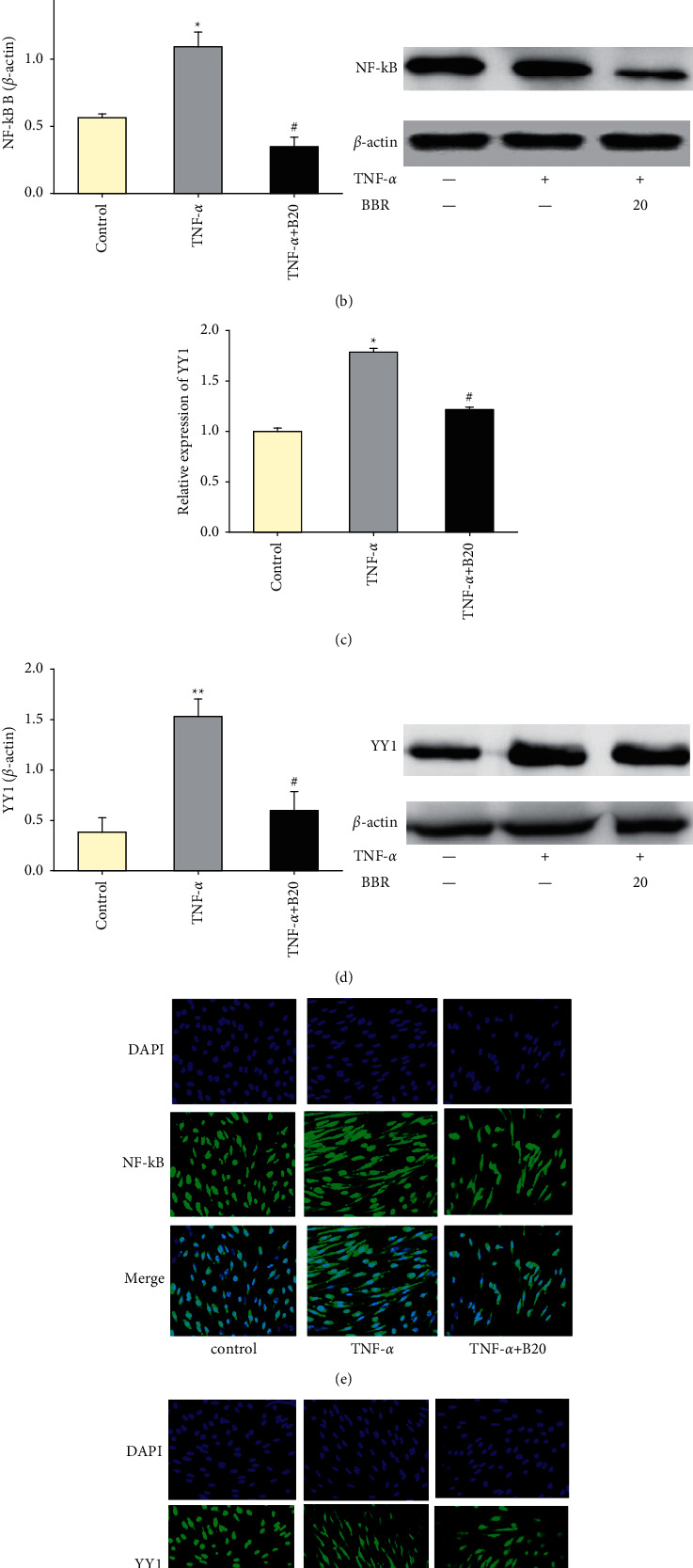
Effect of BBR on the expression of NF-*κ*B and YY1 in TNF-*α*-induced injured HUVECs. (a) mRNA expression of NF-*κ*B p65. (b) mRNA expression of YY1. (c) Expression of NF-*κ*B p65. (d) Expression of YY1. (e) Nuclear translocation of NF-*κ*B p65. (f) Nuclear translocation of YY1. Data are expressed as mean ± SD. *n* = 3. ^*∗*^*P* < 0.05 and ^*∗∗*^*P* < 0.01 compared with the control group. #*P* < 0.05 and ##*P* < 0.01 compared with the TNF-*α* group.

To determine whether the effect of BBR on HUVECs was mediated by decreasing the expressions of NF-*κ*B and YY1, the expressions of mRNA and proteins of NF-*κ*B and YY1 were tested. TNF-*α* treatment increased the mRNA and protein of NF-*κ*B p65 and YY1 (*P* < 0.05 vs. control). As compared with the TNF-*α* group, BBR pretreatment decreased the expressions of mRNA and protein of NF-*κ*B p65 (*P* < 0.05, Figures 4(a) and 4(b)) and YY1 (*P* < 0.05, Figures 4(c) and 4(d)). In addition, TNF-*α* treatment caused an increase of NF-*κ*B and YY1 translocation into the nucleus, while BBR pretreatment attenuated nuclear translocation of NF-*κ*B and YY1 (Figures 4(e) and 4(f)).

### 3.5. Berberine Induces the Activation of AMPK

**Figure 5:**
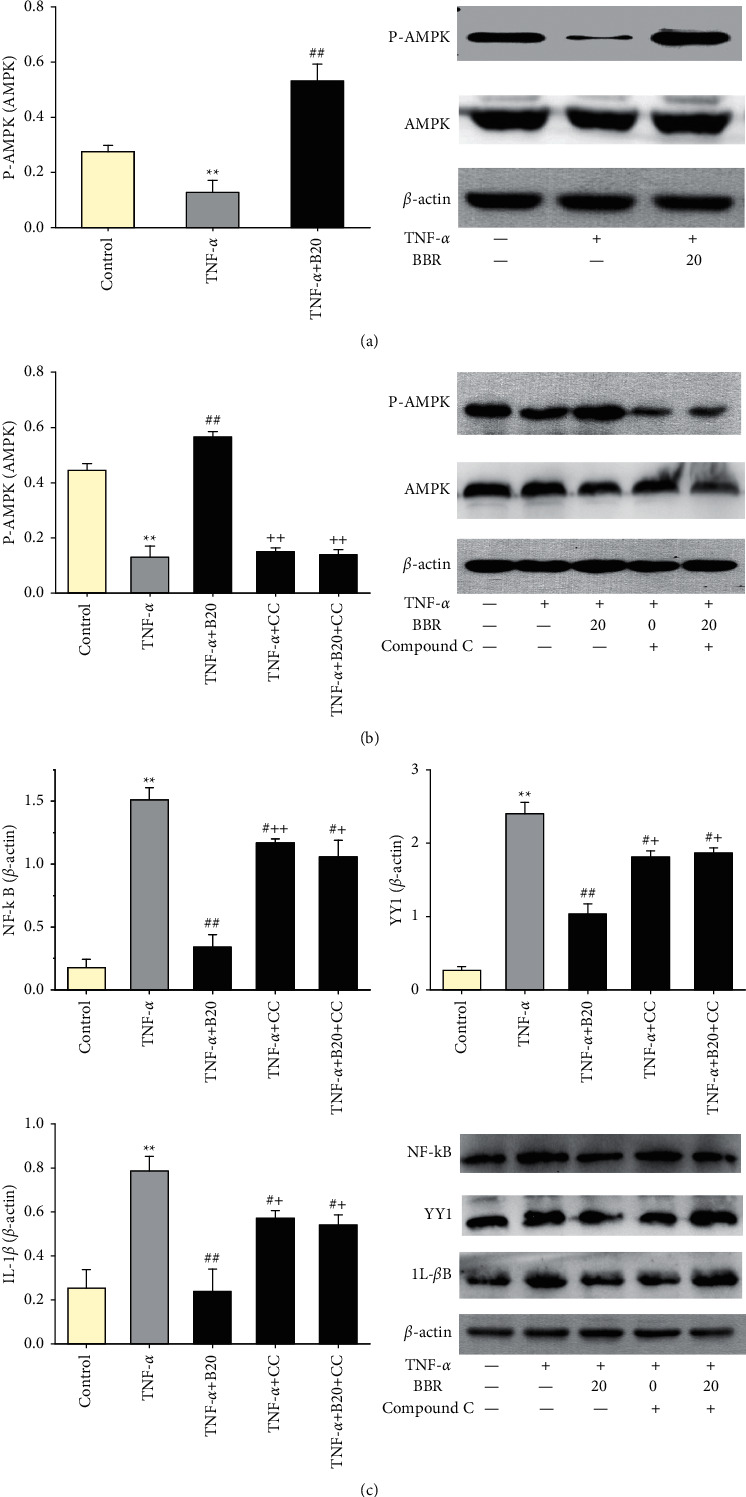
Effect of BBR on the activation of AMPK in TNF-*α*-induced injured HUVECs. **(**a-b) AMPK phosphorylation. (c) Expressions of NF-*κ*B, YY1, and IL-1*β*. Data are expressed as mean ± SD. *n* = 3. ^*∗*^*P* < 0.05 and ^*∗∗*^*P* < 0.01 compared with the control group; #*P* < 0.05 and ##*P* < 0.01 compared with the TNF-*α* group. +*P* < 0.05 and ++*P* < 0.01 compared with the TNF-*α *+ B20 group.

To determine whether the effect of BBR on the inflammation of HUVECs induced by TNF-*α* was associated with AMPK activation, HUVECs were coincubated with compound C (an AMPK inhibitor). As compared with the TNF-*α* group, BBR pretreatment at a concentration of 20 *μ*M activated AMPK phosphorylation in TNF-*α*-induced injured HUVECs (*P* < 0.01, Figure 5(a)). Nevertheless, the effect of BBR was offset when coincubated with compound C (*P* < 0.01, Figure 5(b)). Compound C also lessened the effect of BBR on decreasing the levels of NF-*κ*B, YY1, and IL-1*β* (*P* < 0.05, Figure 5(c)).

## 4. Discussion

This study demonstrated that BBR alleviated endothelial permeability and inflammatory reaction in TNF-*α* induced injured HUVECs. The possible mechanism might be partly associated with activating AMPK and decreasing the expressions of NF-*κ*B and YY1.

Atherosclerosis is a lipid-driven inflammatory disease of the arterial intima in which the balance of proinflammatory and inflammation-resolving mechanisms dictates the final clinical outcome [38]. Increased endothelial permeability initiates a dysregulated transendothelial flux, leading to abnormal deposition of lipids and infiltration of inflammatory cells in the intima, which promotes inflammatory response and endothelial injury [39–41]. In the current study of AS, most pharmaceutical interventions focused on reducing the level of plasma cholesterol and the activation of platelets, such as statins, ezetimibe, PCSK9, clopidogrel, and ticagrelor [42–44], but no effective drug was found to protect against endothelial injury, especially against hyperpermeability and inflammation of endothelial cell. The present study demonstrated that BBR reduced extracellular LDH concentration and EMP numbers in TNF-*α*-induced injured HUVECs, which is consistent with previous studies [45–47]. BBR pretreatment also decreased the overexpression of proinflammatory cytokines, including IL-6, IL-8, and IL-1*β*, in injured HUVECs induced by TNF-*α*, suggesting that BBR has a favorable effect on the inflammatory reaction of endothelial cells induced by TNF-*α*.

AMPK is a stress-activated protein kinase, which serves as a cellular energy sensor [17]. AMPK activation inhibits the inflammatory response in endothelial injury and demonstrates a beneficial effect on atherosclerosis [48, 49]. The present study showed a favorable effect of BBR on TNF-*α*-induced endothelial injury, which was accompanied by AMPK activation. However, compound C, an inhibitor of AMPK, significantly reduced the protective effect of BBR on TNF-*α*-induced endothelial injury. Therefore, our findings indicated that the protective effect of BBR on TNF-*α*-induced endothelial injury was partially associated with AMPK activation.

The NF-*κ*B pathway plays an essential role in inflammation through regulating the genes encoding proinflammatory cytokines (IL-6 and IL-8) and adhesion molecules [50, 51]. Hojo et al. demonstrated that Toll-like receptor- (TLR-) 2-mediated inactivation of NF-*κ*B significantly inhibited the expression of IL-6 and IL-8 [52]. Tang et al. illustrated that the adhesion molecule ICAM-1 was significantly decreased via inhibiting the NF-*κ*B signaling pathway [53]. Moreover, NF-*κ*B activation is closely associated with increased endothelial permeability [54]. YY1, a multifunctional transcription factor, is overexpressed during endothelial injury [55, 56]. The NF-*κ*B/YY1 pathway is one of the inflammatory signaling pathways participating in endothelial injury [22, 23]. However, the association between the AMPK and NF-*κ*B/YY1 pathway is not unclear in our data. Our study illustrated that the activation of AMPK reduced the expression of NF-*κ*B and YY1 in TNF-*α*-induced injured HUVECs, and inhibiting AMPK phosphorylation by compound C increased the expression of NF-*κ*B and YY1. Therefore, the present study indicated that AMPK was upstream of the NF-*κ*B/YY1 signaling pathway, and BBR ameliorated endothelial injury induced by the TNF-*α* via AMPK/NF-*κ*B/YY1 signaling pathway.

There is a limitation in the present study. Only AMPK/NF-*κ*B/YY1 of many downstream pathways of AMPK in HUVECs, which is closely associated with endothelial permeability and inflammation, was examined. Whether BBR has some effects on other downstream pathways of AMPK should be studied in future.

## 5. Conclusions

The present study suggested that BBR protected against TNF-*α*-induced injury in HUVECs via the AMPK/NF-*κ*B/YY1 signaling pathway.

## Figures and Tables

**Table 1:** The primers used in RT-PCR.

Name	Primer sequences
*β*-Actin	F: 5′-GGGTGTGAACCATGAGAAGT-3′
	R: 5′-GACTGTGGTCATGAGTCCT-3′

NF-*κ*B	F: 5′-GGGATGGCTTCTATGAGGCT-3′
	R: 5′-CTGACTGATAGCCTGCTCCA-3′

YY1	F: 5′-GTCTGTGCAGAATGTGGCAA-3′
	R: 5′-TGTGCGCAAATTGAAGTCCA-3′

F: forward primer; R: reverse primer.

## Data Availability

The datasets in this study can be obtained from the corresponding author upon reasonable request at shidazhuo@yeah.net.

## Abbreviations

AS: Atherosclerosis
TNF: Tumor necrosis factor
AMPK: Adenosine 5′-monophosphate activated protein kinase
NF-*κ*B: Nuclear factor kappa B
YY1: Yin Yang 1
BBR: Berberine
HUVECs: Human umbilical endothelial cells
LDH: Lactic dehydrogenase
EMPs: Endothelial microparticles
ELISA: Enzyme-linked immunosorbent assay
RT-PCR: Real-time polymerase chain reaction
CC: Compound C
LDL: Low-density lipoprotein
T2DM: Type-2 diabetes mellitus
TLR: Toll-like receptor.

## Supplementary Materials

Supplementary Materials Supplementary materials: raw data of western blot.
