# Supplementary material for: Berberine Protects against TNF-α-Induced Injury of Human Umbilical Vein Endothelial Cells via the AMPK/NF-κB/YY1 Signaling Pathway
Source: Evid Based Complement Alternat Med. 2021 Dec 31;2021:6518355. doi: 10.1155/2021/6518355 (PMC8741384; doi:10.1155/2021/6518355)

The raw data of WB are as follows. Because the methodsof running WB are different by two personal respectively, the picture are differentlypresented too.


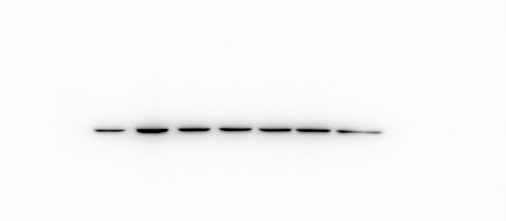

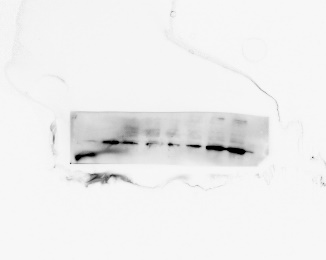

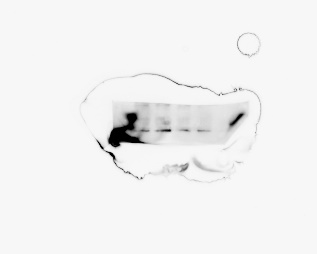


Figure s1 IL-1β


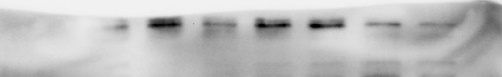

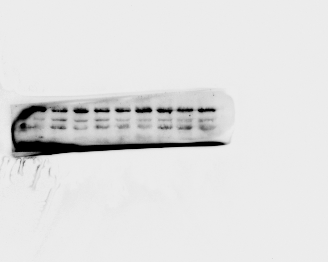

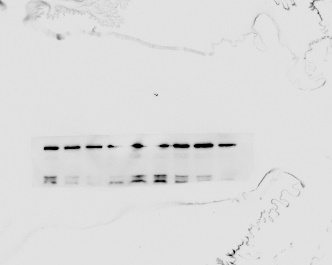


Figure s2 IL-1βafteradding compound C


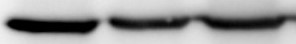

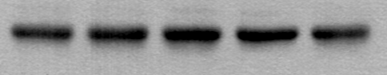


Figure s3β-actin


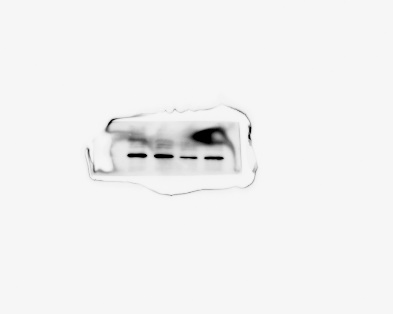

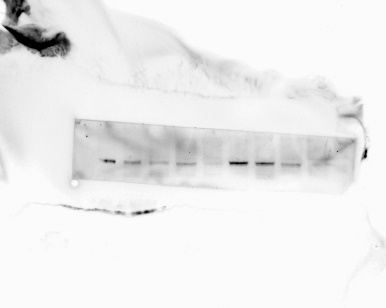


Figure s4 NF-KB


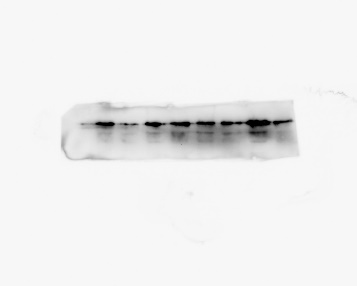

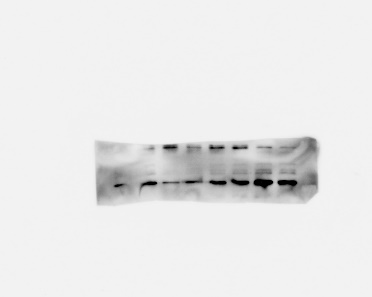

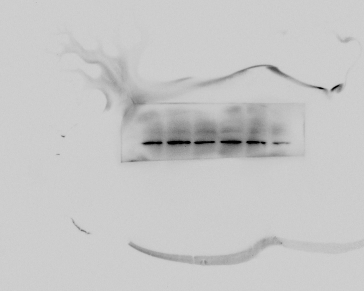


Figure s5 NF-KBafter adding compound C


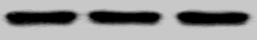

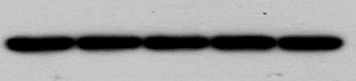


Figure s6 β-actin


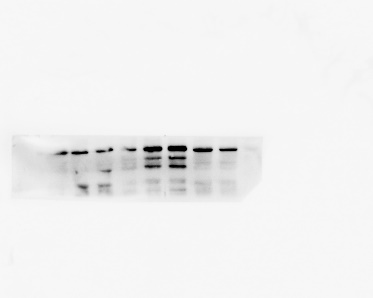

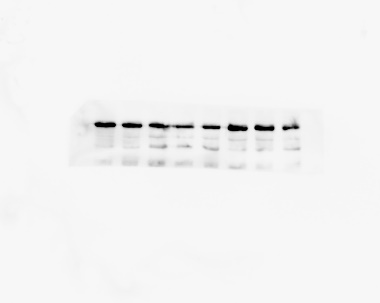


Figure s7 YY1


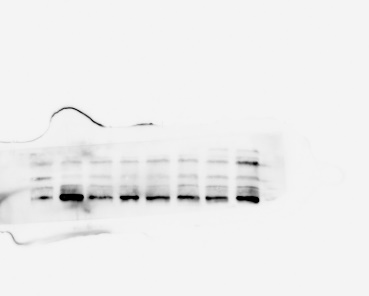

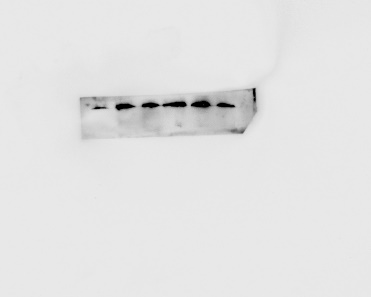

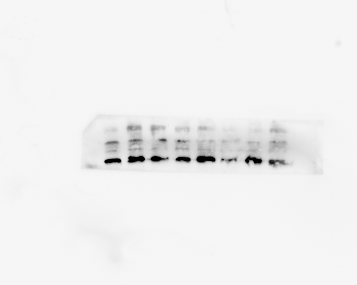


Figure s8 YY1after adding compound C


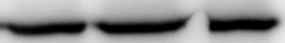

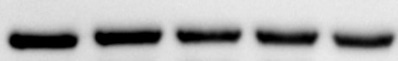


Figure s9 β-actin


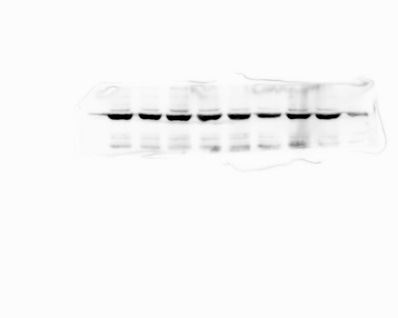

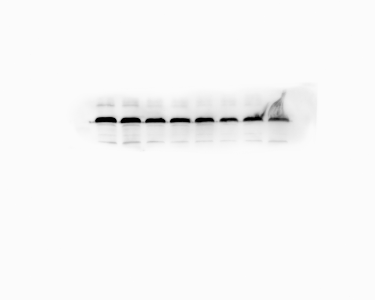

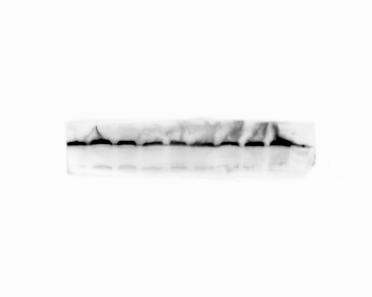


Figure s10 AMPK


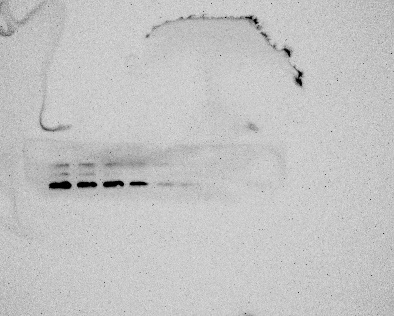


Figure s11 Phosphorylation of AMPK


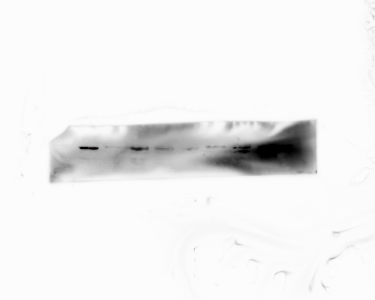

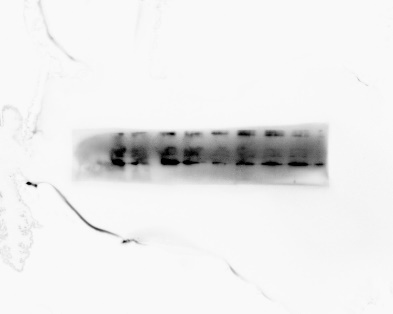


Figure s12 AMPK after adding compound C


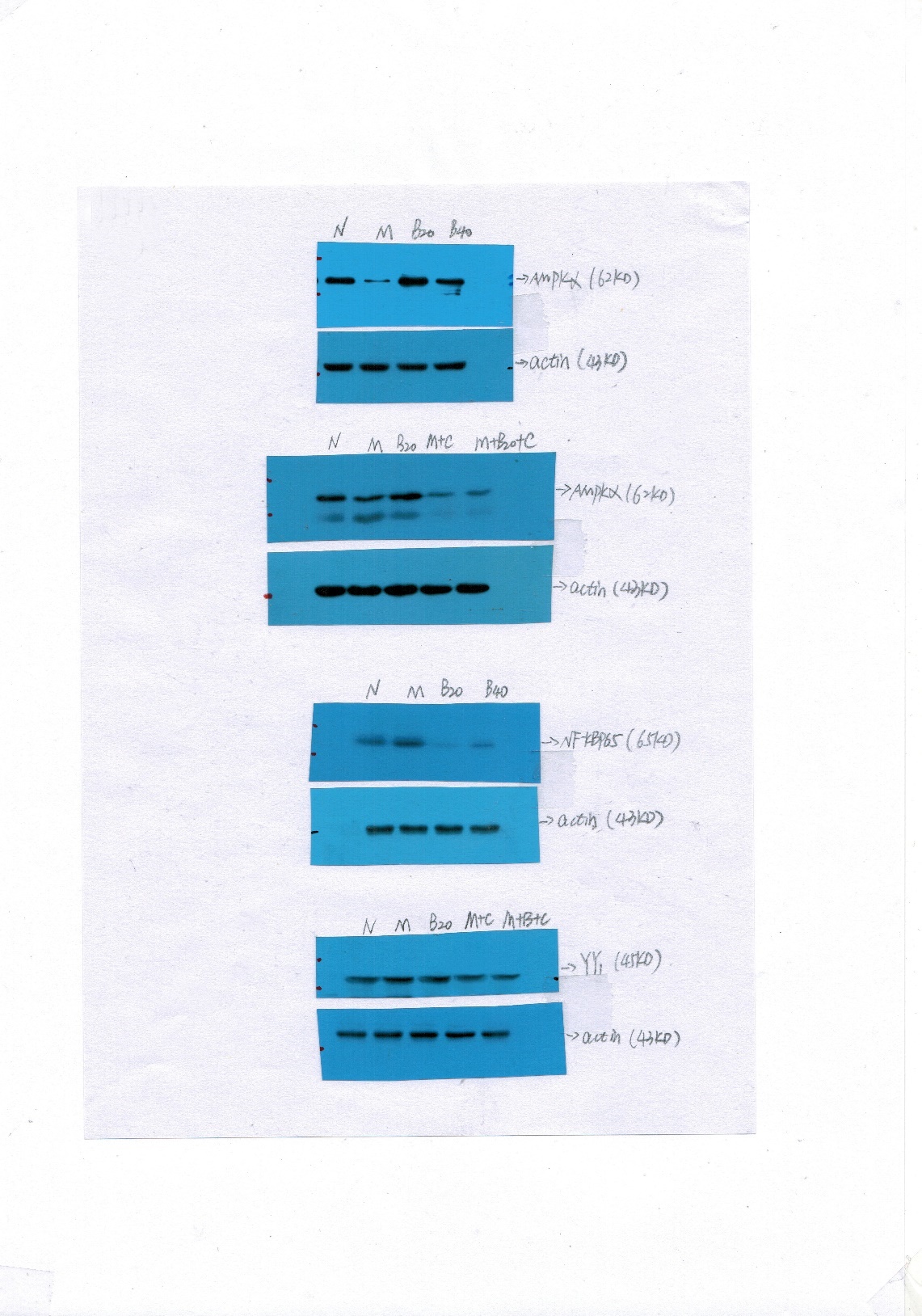


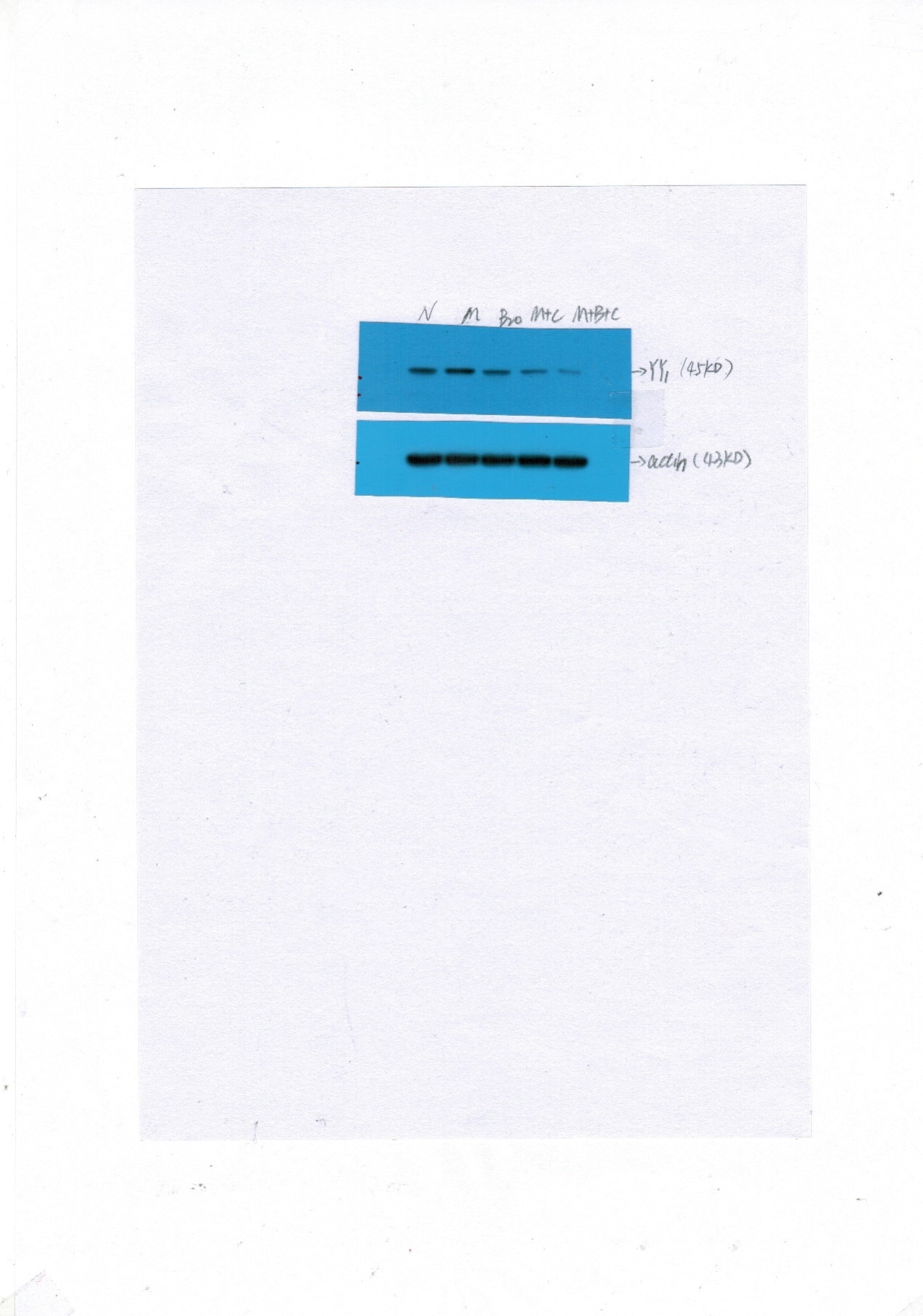


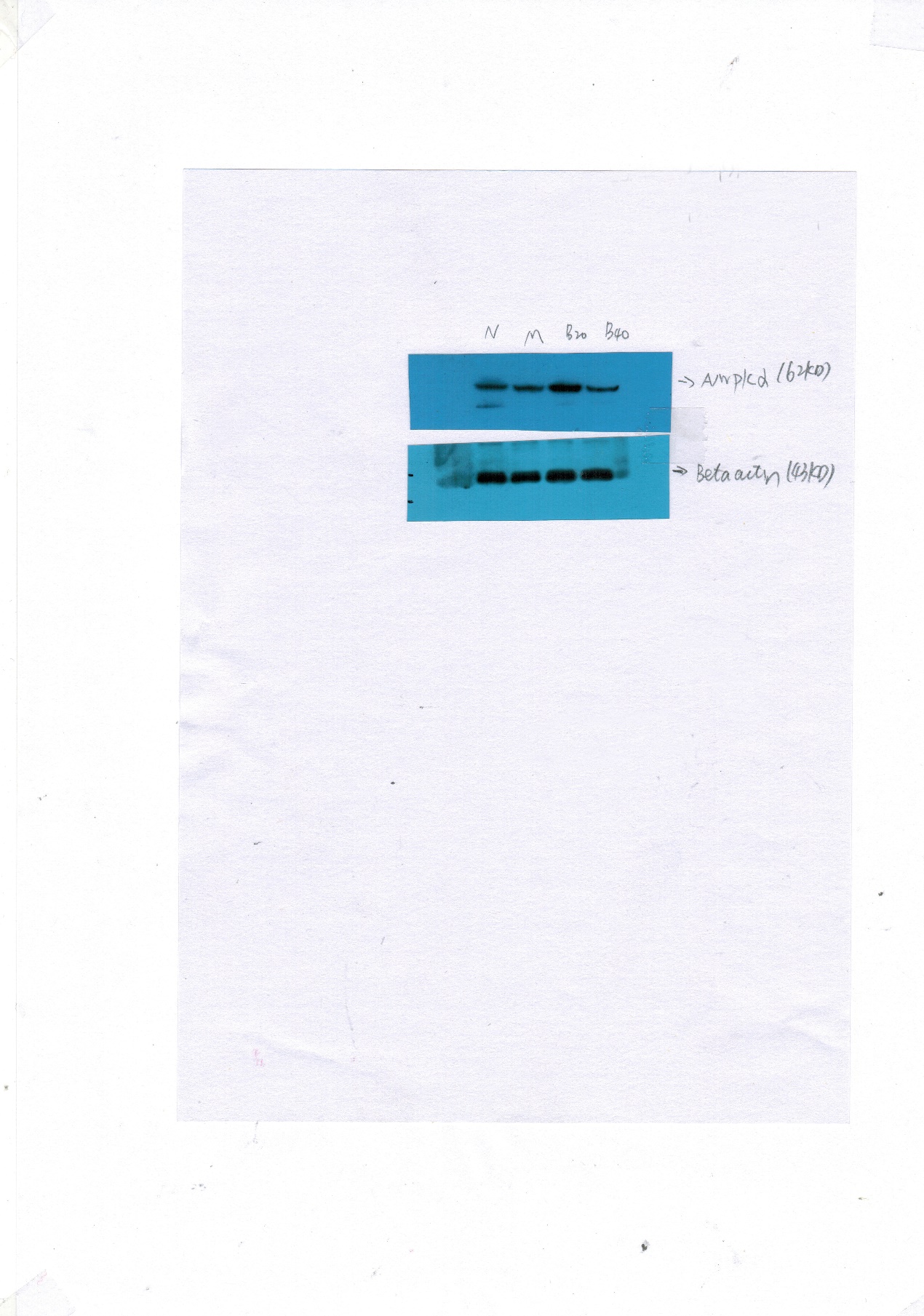

Supplement: Supplementary Materials — raw data of western blot. [file 6518355.f1.docx]
